# Kidney thrombotic microangiopathy in lupus nephritis: Impact on treatment and prognosis

**DOI:** 10.1177/09612033221106301

**Published:** 2022-06-01

**Authors:** David Massicotte-Azarniouch, Elizabeth Kotzen, Sarah Todd, Yichun Hu, Susan L. Hogan, Koyal Jain

**Affiliations:** 1 Division of Nephrology and Hypertension, Department of Medicine, 6797 University of North Carolina at Chapel Hill, Chapel Hill, NC, USA; 2 Department of Medicine and Pediatrics, 6797 University of North Carolina at Chapel Hill, Chapel Hill, NC, USA

**Keywords:** lupus nephritis, thrombotic microangiopathy, remission, end-stage kidney disease

## Abstract

**Introduction:**

Lupus nephritis (LN) may present with thrombotic microangiopathy (TMA) on kidney biopsy, the impact of which on outcomes is unclear. This study examined the prognostic importance of LN with TMA on kidney biopsy, including response to therapy and long-term outcomes.

**Methods:**

We conducted a single-center, retrospective study of all cases of LN with concomitant TMA on kidney biopsy in the Glomerular Disease Collaborative Network database. Controls were individuals with LN without TMA matched to cases based on demographic and clinical variables. Outcomes were remission at 6- and 12-months, end-stage kidney disease (ESKD) and death. Logistic regression and Cox proportional hazards models were used to ascertain the risks for outcomes, with adjustment for serum creatinine and proteinuria.

**Results:**

There were 17 cases and 28 controls. Cases had higher creatinine, higher proteinuria and greater chronicity on biopsy at baseline compared to controls. The rates of remission at 6-months and 12-months were similar between cases and controls (6-months 53.9% vs 46.4%, adjusted OR 2.54, 95% CI 0.48, 13.37; 12-months 53.9% vs 50.0%, adjusted OR 2.95, 95% CI 0.44, 19.78). Cases were at greater risk for ESKD in univariate analysis (HR 3.77; 95% CI 1.24, 11.41) but not when adjusting for serum creatinine and proteinuria (HR 2.20; 95% CI 0.63, 7.71). There was no significant difference in the risk of death between cases and controls.

**Conclusion:**

Lupus nephritis with renal TMA likely responds to therapy similarly to those without TMA; risk for ESKD is not significantly increased, although the influence of renal function and proteinuria in larger samples is needed.

## Introduction

Lupus nephritis (LN) is a major complication of systemic lupus erythematosus (SLE), occurring in 20–60% of patients with SLE, depending on race and ethnicity.^1–3^ It is characterized by immune complex deposition within the glomerulus, leading to inflammation and endothelial damage, ultimately leading to end-stage kidney disease (ESKD) in up to 10% of patients.^4,5^ While the location and extent of glomerular inflammatory cell proliferation is the major basis for the current histopathologic classification of LN, vascular lesions may also be found, some of which may have prognostic implications.^6^

Thrombotic microangiopathy (TMA) describes syndromes which share pathologic features of vascular damage within the walls of arterioles and capillaries leading to microvascular thrombi. In the kidney microvasculature, TMA results in compromised blood flow and glomerular capillary thrombi formation, leading to acute kidney injury, and remodeling changes when the insult is persisting or recurring.^7^ Thrombotic microangiopathy is one of the various vascular lesions seen in lupus nephritis, observed in 8-17% of lupus nephritis biopsies.^6,8^ When patients manifest LN with concomitant TMA on biopsy, this has been associated with adverse kidney outcomes, however it is unclear if this is simply due to worse kidney function and more chronic damage at presentation than those without TMA.^9–12^ Whether or not patients with LN and TMA respond as well to standard LN therapies, and what impact this may have on long term outcomes, is unclear.

This study aimed to describe the clinical characteristics, treatments, and outcomes of patients with LN and kidney TMA, and to compare the rates of remission as well as the kidney prognosis to patients with LN without TMA.

## Methods

### Design and setting

We conducted a retrospective study of individuals (pediatric and adult) with concomitant biopsy-confirmed LN and TMA. The study cohort was derived from the Glomerular Disease Collaborative Network (GDCN) registry at the University of North Carolina in Chapel Hill. The GDCN is a prospectively collected, longitudinal follow-up registry of patients with biopsy confirmed glomerular disease which patient level data (demographics, clinical variables, biological specimens) over the course of their disease.^13^ This study was approved by our center’s Institutional Review Board, and informed consent was waived due to its retrospective nature.

Cases

In the GDCN, diagnoses are coded based on the pathological report. Therefore, all individuals with a LN diagnosis are coded accordingly. GDCN was searched from 1980 to 2020; cases were defined as an individual with a kidney biopsy showing LN with the presence of TMA affecting the vessels and/or glomeruli based on a keyword search within the pathology report looking for a sub-diagnosis of TMA. All flagged individuals were verified through review of the biopsy report to confirm concomitant LN with TMA. We excluded individuals with end-stage kidney disease [ESKD] (at least 3 months of regular dialysis) at time of the biopsy. We also excluded individuals with missing key data (inability to determine the remission status at 6- and 12-months due to loss to follow-up).

### Controls

Controls were individuals with LN (captured using GDCN as described above) without TMA on their biopsy. This yielded a source population of 601 potential controls. We then restricted the control cohort to those who had a medical chart available for review and who shared similar characteristics to our cases. After observing that all cases were between the ages of 10 and 39, were either of non-Hispanic black, non-Hispanic white, or Hispanic race/ethnicity and the earliest case was in 1999, we next restricted the control cohort to those who shared these demographic characteristics with our cases. We were then left with a cohort of 278 controls. Using this subset, controls were frequency matched to cases based on: (a) sex (male vs female); (b) age group at biopsy (10–19 vs 20–29 vs 30–39); (c) race (black vs non-black); (d) LN class (proliferative class III/IV vs other, based on ISN/RPS classification); (e) biopsy year (1995–2005 vs 2006–2020); and (f) baseline kidney function (glomerular filtration rate [GFR] <30 ml/min vs ≥30 ml/min). These choices were determined prior to analysis, and based on what we felt were clinically important variables within the constraints imposed by our sample size.

### End points

Our primary end points were remission at 6 and 12 months after index biopsy (histopathologic diagnosis of LN). Remission was defined as either complete or partial based on urine protein-creatinine ratio (Upcr) and serum creatinine (SCr). Complete remission was defined as Upcr < 0.5 g/g and normal SCr. Partial remission was defined as a Upcr decrease of ≥50% from baseline where Upcr <1 g/g if baseline was <3 g/g or Upcr <3 g/g if baseline Upcr >3 g/g, and SCr improved or no worse than baseline. Secondary outcomes were complete remission at 6 months, complete remission at 12 months, time to ESKD, and time to death.

### Statistical analysis

Descriptive statistics included means with standard deviations (SD) or medians with interquartile ranges (IQR) for continuous measures, and counts with percentages for categorical variables. Comparisons between cases and controls were evaluated using student t-tests, wilcoxon rank tests, chi-square test or Fischer’s exact tests, where appropriate. The baseline characteristics determined for each study individual were: age, sex, race, LN class, interstitial fibrosis and tubular atrophy (IFTA) score on index biopsy, percent crescents on index biopsy, and laboratory values at time of biopsy (SCr, Upcr, serum albumin, C3, C4, hemoglobin [Hb], platelets, anti-phospholipid antibody [APLA] presence yes/no). We also determined treatments used for induction therapy (during the first 6 months), the presence of a thrombotic complication (arterial or venous) during the initial presentation and if there was patient non-adherence (either not showing up for medical follow-up or mention of medication non-adherence) during follow-up. We also provided a description of the evolution of kidney function over time after index biopsy in both cases and controls.

Logistic regression was used to calculate odds ratios [OR] for remission. Time-to-event was calculated from day 0 (index biopsy date) to ESKD, death or last known follow-up. Unadjusted Kaplan-Meier curves were plotted and Cox proportional hazards models were used to calculate hazards ratios [HR] and 95% confidence intervals (CI) for ESKD and for death. Due to the small sample size, we limited the number of covariates used in adjusted models to no more than 2 to have an approximate event to variable ratio of 5. After frequency matching, imbalances were observed between the cases and controls regarding baseline SCr, and Upcr, therefore these variables were controlled for in our final adjusted models given their prognostic importance in LN. We also performed the following sensitivity analyses to more thoroughly examine the ESKD risk associated with LN and TMA: (1) looking at ESKD risk after adjusting for IFTA; and (2) looking at ESKD risk in the population restricted to index biopsy in the years 2011–2020 given the difference in follow-up duration between cases and controls. P-values < .05 were considered statistically significant. All analyses and plots were done using SAS software (Version 9.4 SAS Institute, Cary, NC).

## Results

**Figure 1.**
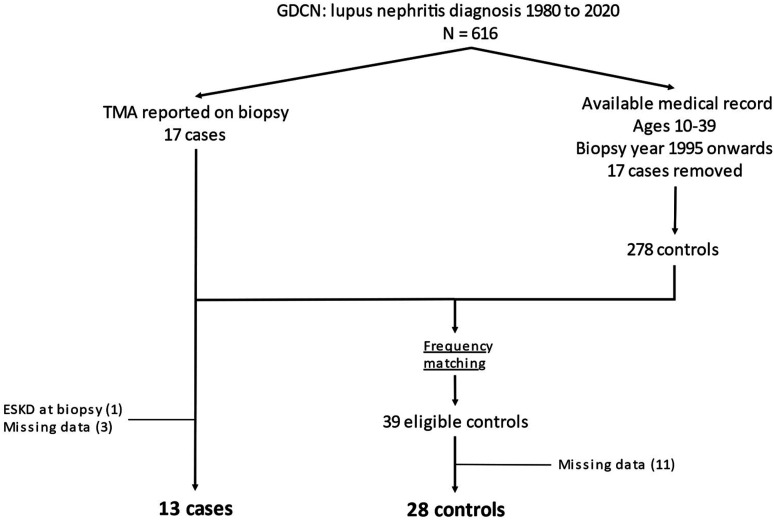
Study cohort creation flow chart. GDCN: Glomerular Disease Collaborative Network; TMA: thrombotic microangiopathy; ESKD: end-stage kidney disease.

Out of a source population of 616 patients in GDCN, there were 17 cases of LN with concomitant kidney TMA and 39 controls after applying our selection criteria (Figure 1). Forty-six percent of cases, compared to 32% of controls, were diagnosed between 2011 and 2020. Four cases and 11 controls were excluded (1 case had ESKD at time of biopsy, 3 cases and 11 controls had missing data), leaving a final study population of 13 cases and 28 controls (Figure 1). Cases had higher SCr and Upcr, lower Hb and platelets, more presence of LN class IV and greater chronicity on biopsy than controls. Cases had a non-statistically significant greater presence of a thrombotic complication during their presentation (46.2% vs 25.0%, respectively, *p* = .28) and use of cyclophosphamide during induction therapy (76.9% vs 57.1% respectively, *p* = .30). Use of anti-thrombotic agents and APLA positivity at time of biopsy was similar between the 2 groups (Table 1). There was 1 case and 1 control who had possible anti-phospholipid syndrome at time of the diagnosis of LN. The median (IQR) follow-up time was 1.9 (0.8–4.3) years for cases and 8.6 (2.9–10.5) years for controls.

**Table 1.** Baseline characteristics of cases and controls.

	Cases (*n* = 13)	Controls (*n* = 28)	*P*-value^†^
Baseline characteristics			
Age; Median (IQR)	22 (21–28)	26.5 (22.0–31.5)	.22
Pediatric, *n* (%)^a^	2 (15.4)	0 (0)	<.0001
Sex, female; *n* (%)	8 (61.5)	25 (89.3)	.08
Race, black; *n* (%)^b^	11 (84.6)	22 (78.6)	1.00
Year of biopsy; *n* (%)			
1995–2000	2 (15.4)	1 (3.6)	
2001–2010	5 (38.5)	18 (64.3)	.15
2011–2020	6 (46.2)	9 (32.1)	
SCr; Median (IQR)	4.7 (2.6–5.9)	1.1 (0.7–2.9)	.0007
Upcr; Median (IQR)	4.7 (2.9–7.4)	2.2 (1.6–3.3)	.03
Serum albumin; Median (IQR)	2.7 (1.8, 3.1)	2.6 (2.1–3.1)	.79
Lupus nephritis class; *n* (%)			
II	1 (7.7)	3 (10.7)	
III	0 (0)	8 (28.6)	.0194
IV	10 (76.9)	8 (28.6)	
V	2 (15.4)	9 (32.1)	
IFTA score on biopsy; *n* (%)			
None-mild (0–1)	5 (38.5)	23 (82.1)	.0102
Moderate-severe (2–3)	8 (61.5)	5 (17.9)	
Percent of glomeruli with crescents			
0%	8 (61.5)	17(60.7)	
1–49%	3 (23.1)	8 (28.6)	1.00
>50%	2 (15.4)	3 (10.7)	
C3 level at biopsy; Median (IQR)	49 (24–7)	67 (0.1–86)	.33
C4 level at biopsy; Median (IQR)	7 (0.1–8)	9 (0.1–20)	.08
Hb level at biopsy; Median (IQR)	7.8 (6.7–8.2)	10.8 (9.75–12)	<.0001
PLT level at biopsy; Median (IQR)	73 (53–130)	210 (114–299.5)	.0069
Thrombosis on presentation; *n* (%)^c^	6 (46.2)	7 (25.0)	.28
Anti-thrombotic agent use; *n* (%)	6 (46.2)	11 (39.3)	.78
Cytoxan use at induction; *n* (%)	10 (76.9)	16 (57.1)	.30
APLA positivity at biopsy; *n* (%)^d^	4 (30.8)	7 (30.4)	1.00

Albumin, C3 and C4 level were not done for 1 individual and APLA positivity for five.

^†^P values were calculated by Fisher Exact test for categorical variables and Wilcoxon Two Sample tests for continuous variables.

^a^The pediatric controls obtained from frequency matching were among the 11 controls with missing outcomes data who were excluded from the study.

^b^Non-black race included individuals of Hispanic ethnicity.

^c^For cases, 3 received aspirin and 3 anticoagulation. For controls, 8 received aspirin and 3 anticoagulation.

^d^APLA positivity was defined as anti-β2-glycoprotein or anti-cardiolipin level above upper limit reference range (IgM or IgG) or lupus anticoagulant assay interpreted as positive.

APLA: antiphospholipid antibody; Hb: hemoglobin; IFTA: interstitial fibrosis and tubular atrophy; IQR: interquartile range; PLT: platelet; SCr: serum creatinine (md/dL); Upcr: urine protein to creatinine ratio (g/g).

### Description of cases

**Figure 2.**
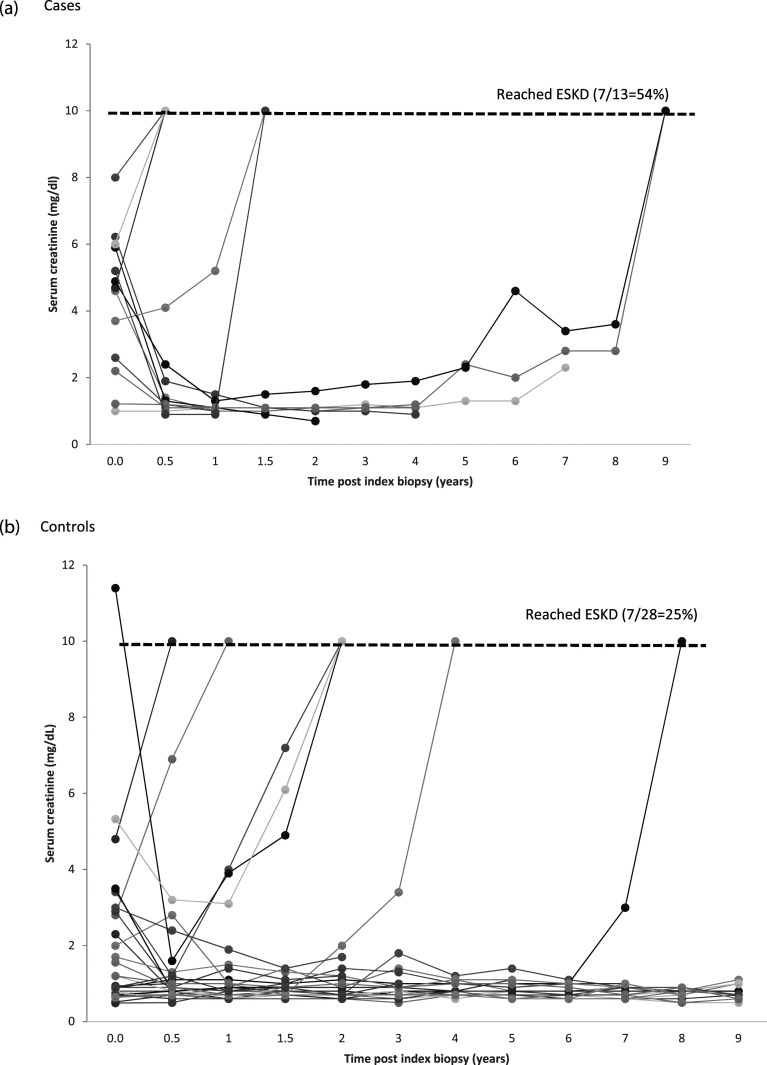
Trends of serum creatinine values after index biopsy in cases and controls. (a) Cases. (b) Controls. The line representing ESKD means that a given individual reached ESKD during their follow-up. It does not necessarily mean that the serum creatinine at last follow-up was 10 mg/dL. ESKD: end-stage kidney disease.

Many cases had elevated SCr at presentation but still responded to treatment to achieve remission (Figure 2, Table 2). Although cases seemed to present with worse kidney function, the overall patterns of evolution of kidney function were similar between cases and controls; some deteriorated quickly to ESKD, most improved and achieved remission, while some deteriorated after initial improvement (Figure 2). Cases were treated with standard regimens, mostly consisting of cyclophosphamide ± mycophenolate during the first 6 months and then mycophenolate maintenance therapy. Five out of 13 cases (38.5%) also received plasmapheresis and 1 (7.7%) received eculizumab (Table 2). Plasmapheresis was mostly initiated for initial suspicion of thrombotic thrombocytopenic purpura, but in 1 case it was specifically for severe lupus manifestations (LN and kidney TMA) with positive APLA. Eculizumab was given for initial suspicion of complement-mediated kidney TMA but was stopped after genetic testing returned negative. Four out of the 5 with plasmapheresis achieved remission, 2/5 developed ESKD, and the 1 patient treated with eculizumab achieved remission with normal SCr on last follow-up. None of the cases had any obvious cause for kidney TMA other than active lupus nephritis. Overall, 24 individuals were deemed non-adherent (7 cases [53.9%] and 17 controls [60.7%], *p* = .74).

**Table 2.** Description of cases at time of biopsy, treatments received and outcomes.

Patient	Biopsy year	Sex	Age	Race	SCr	Upcr	APLA	LN Class	IFTA	% cres	Induction^a^	Maintenance^b^	Remission^c^	Status at last f/u
1	1999	F	22	B	3.7	2.9	0	4	Sev	0	Pred-HCQ	MMF-CNI	No	ESKD Died
2	2000	M	11	B	8.0	7.8	0	4	Mod	0	CYC-PP	-	No	ESKD Alive
3	2002	F	27	B	2.2	1.5	0	2	Mod	0	AZA	MMF-AZA	No	ESKD Alive
4	2005	M	13	B	4.6	3.8	0	4	Mild	5	CYC-MMF	MMF	Yes	No ESKD Died
5	2005	F	22	B	4.7	6.2	1	4	Mod	50	CYC-MMF-AZA	MMF-AZA	No	ESKD Died
6	2005	M	28	B	6.0	7.4	1	4	Mod	0	CYC	-	No	ESKD Died
7	2006	F	33	B	5.2	4.7	0	4	Mild	0	CYC-MMF	CYC-MMF-AZA	Yes	ESKD Died
8	2011	M	22	B	4.9	4.4	0	4	Mod	10	CYC-PP-MMF	MMF	Yes	ESKD Alive
9	2013	F	21	B	1.0	0.9	0	5	None	0	MMF	MMF	Yes	No ESKD Alive SCr 2.3
10	2016	F	27	B	6.2	5.3	1	4	Mod	30	CYC-PP-MMF	MMF-AZA	Yes	No ESKD Alive SCr 0.9
11	2016	F	37	H	1.2	7.4	0	4	Mild	0	CYC-PP-MMF-AZA	AZA-CNI	Yes	No ESKD Alive SCr 1.2
12	2019	F	29	W	5.9	12.8	1	4	None	77	CYC-MMF-CNI	MMF-CNI	Yes	No ESKD Alive SCr 0.7
13	2020	M	21	B	2.6	0.8	0	5	Mod	0	CYC-PP-ECU-MMF	MMF	Yes	No ESKD Alive SCr 1.0

All patients received prednisone and hydroxychloroquine.

^a^Refers to medications received for treatment of lupus nephritis during the first 6 months after index biopsy.

^b^Refers to medications received for treatment of lupus nephritis beyond the first 6 months of therapy (does not include re-induction for treatment of flares).

^c^Either complete or partial remission at 6 or 12 months.

APLA: anti-phospholipid antibodies; AZA: azathioprine; B: black; cres: crescents; CNI: calcineurin inhibitor; CYC: cyclophosphamide; ECU: eculizumab; ESKD: end-stage kidney disease; H: Hispanic; IFTA: interstitial fibrosis tubular atrophy; LN: lupus nephritis; MMF: mycophenolate mofetil; Mod: moderate; PP: plasmapheresis; SCr: serum creatinine (mg/dL); Sev: severe; Upcr: urine protein to creatinine ratio (g/g); W: white.

### Remission at 6- and 12-months

Among cases, 53.9% achieved remission at 6-months and at 12-months (complete remission 23.1% at 6 months and 30.8% at 12 months). Among controls, 46.4% achieved remission at 6 months and 50% at 12 months (complete remission 25% at 6 months and 35.7% at 12 months) (Table 3). There was no statistically significant difference in the unadjusted odds for achieving complete or partial remission between cases and controls at 6 months nor at 12 months (OR 1.35 95% CI 0.36, 5.04; and OR 1.17 95% CI 0.31, 4.36 respectively). Although models adjusted for SCr and Upcr levels also showed no statistically significant difference in the odds for remission between the two groups, there was a consistently higher point estimate for the odds for remission in cases at 6 and 12 months (OR 2.54 95% CI 0.48, 13.37; and 2.95 95% CI 0.44, 19.78 respectively) (Table 4).

**Table 3.** Frequency of outcomes in cases compared to controls.

	Cases (*n* = 13)	Controls (*n* = 28)	*P*-value
Follow-up, years; median (IQR)	1.9 (0.8–4.3)	8.6 (2.9–10.5)	.0081
Non-adherent; *n* (%)	7 (53.8)	17 (60.7)	.74
Complete or partial remission at 6 mo; *n* (%)	7 (53.9)	13 (46.4)	.74
Complete remission	3 (23.1)	7 (25.0)	1.00
Complete or partial remission at 12 mo; *n* (%)	7 (53.9)	14 (50.0)	1.00
Complete remission	4 (30.8)	10 (35.7)	1.00
ESKD; *n* (%)	7 (53.9)	7 (25.0)	.09
Death; *n* (%)	5 (38.5)	8 (28.6)	.72

ESKD: end-stage kidney disease; IQR: interquartile range.

**Table 4.** Odds ratios and hazards ratios for outcomes in cases compared to controls.

Outcomes	OR/HR (95% CI) for cases vs controls	
Complete or partial remission at 6 months	OR unadjusted	1.35 (0.36–5.04)
	OR adjusted for SCr	2.28 (0.45–11.65)
	OR adjusted for Upcr	1.76 (0.41–7.57)
	OR adjusted for SCr & Upcr	2.54 (0.48–13.37)
Complete or Partial remission at 12 months	OR unadjusted	1.17 (0.31–4.36)
	OR adjusted for SCr	3.20 (0.50–20.37)
	OR adjusted for Upcr	1.03 (0.25–4.27)
	OR adjusted for SCr & Upcr	2.95 (0.44–19.78)
ESKD	HR unadjusted	3.77 (1.24–11.41)
	HR adjusted for SCr	2.30 (0.76–6.92)
	HR adjusted for Upcr	3.39 (1.06–10.86)
	HR adjusted for SCr & Upcr	2.20 (0.63–7.71)
	HR adjusted for IFTA	2.09 (0.61–7.23)
Death	HR unadjusted	1.31 (0.40–4.33)
	HR adjusted for SCr	0.80 (0.22–2.91)
	HR adjusted for Upcr	1.16 (0.33–4.09)
	HR adjusted for SCr & Upcr	0.80 (0.20–3.17)

ESKD: end-stage kidney disease; HR: hazards ratio; IFTA: interstitial fibrosis and tubular atrophy; LN: lupus nephritis; OR: odds ratio; SCr: serum creatinine; TMA: thrombotic microangiopathy; Upcr: urine protein to creatinine ratio.

### ESKD and death

End-stage kidney disease occurred in 14 individuals, 7 (53.9%) cases and 7 (25.0%) controls (*p* = .09). Thirteen individuals died, 5 (38.5%) cases and 8 (28.6%) controls (*p* = .72). Causes of death for cases were sepsis (3), myocardial infarction (1) and unknown (1), and for controls they were SLE (1), endocarditis (1), pulmonary embolism (1), sudden cardiac arrest (1), sub-dural hematoma (1), pancreatic cancer (1) and unknown (2). Although unadjusted analysis suggested worse kidney survival in cases (HR for ESKD 3.77 95% CI 1.24, 11.41), the risk for ESKD was not statistically significantly greater after adjusting for SCr and Upcr (adjusted HR 2.20 95% CI 0.63, 7.71) (Table 4). When adjusting for IFTA, the HR for ESKD was also not statistically significant (2.09 95% CI 0.61, 7.23). When restricting the study period from 2011 to 2020, there were 6 cases and 9 controls with a median (IQR) follow-up time of 3.9 (2.3–6.5) years compared to 7.7 (2.7–8.2) years, respectively; 1 case (16.7%) and 2 controls (22.2%) went on to ESKD. For death, there was no statistically significant difference in the unadjusted overall patient survival (HR 1.31 95% CI 0.40, 4.33) nor when adjusted for SCr and Upcr (adjusted HR 0.80 95% CI 0.20, 3.17) (Table 4).

## Discussion

In this single-center, retrospective study examining rates of response to therapy and prognosis from LN with concomitant TMA on kidney biopsy, we found that the presence of TMA was not associated with worse outcomes. There was no significant difference in the odds of achieving remission at 6-months (52.9% vs 46.4% for cases vs controls, unadjusted OR 1.35 [95% CI 0.36, 5.04]) nor at 12-months (53.9% vs 50.0% for cases vs controls, unadjusted OR 1.17 [95% CI 0.31, 4.36]). This was despite LN TMA cases having higher baseline SCr, proteinuria and chronicity on biopsy compared to controls. When adjusting for kidney function at baseline similar results were found. End-stage kidney disease occurred more frequently in cases (53.9% vs 25.0% for cases vs controls), however there was no statistically significant difference in the risk for ESKD when adjusting for important clinical variables SCr, Upcr and IFTA (adjusted HR 2.20 95% CI 0.63, 7.71).

We found only 17 cases of kidney TMA concomitant with LN from our GDCN registry out of 616 LN patients accrued over a 30-year period (2.8%). Other studies have found occurrence rates ranging from 3.5-17%.^6,8,12^ Since our capture of cases was based on keyword finding in pathology reports and not a systematic revision of every single biopsy report, it is possible that some cases were missed. Also, our capture is dependent on a pathologist recognizing the lesion. TMA has been more readily recognized as a kidney lesion in the last 10 years or so meaning pathologists may be more likely to diagnose this in the modern era compared to the early 1990s or 2000s. Indeed, nearly 50% of our cases were diagnosed between 2010 and 2020. Due to better recognition, the occurrence of LN with TMA may be more common in the current era.

The cases of LN with TMA in our study had more severe presentation, as evidenced by more impaired kidney function, greater proteinuria and more chronicity on biopsy compared to controls. Despite this, they were able to achieve similar remission rates as in controls. Even cases who presented with very elevated SCr often saw marked improvement in their kidney function with treatment. Pattanashetti et al also examined treatment response and showed that patients with TMA did not respond as well as those without TMA. However, this may have been due to an unusually high remission rate in their non-TMA group (79% for LN non-TMA vs 50% for LN-TMA) and/or the fact that remission was only assessed at 6 months.^14^ The remission rates for LN with TMA in our study were similar to those of the same group in the Pattanashetti study, and also similar to rates usually seen in LN studies.^15–18^ Li et al showed no difference in remission rates at 12-months in LN with TMA compared to without.^12^ Therefore, TMA does not necessarily portend more refractory disease. Interestingly, when adjusting for baseline kidney function and proteinuria, we found a trend towards an increased odds for remission in LN with TMA. This may be due to clinicians treating such patients more aggressively (longer duration or higher doses of immunosuppression) or providing better patient counseling, thus contributing to better treatment outcomes.

Cases of LN with TMA were not at significantly greater risk for ESKD when adjusting for clinical and histological variables. In our unadjusted analyses, ESKD occurred faster and more frequently in cases, with a HR of nearly 4. However, adjusting for baseline SCr and for degree of chronicity on biopsy nearly halved this risk and there was no longer a statistically significant association. Other studies of LN with TMA have shown an association with worse kidney survival, also in the context of worse baseline kidney function and greater chronicity on biopsy.^9–12^ Kidney TMA may manifest as arteriolar or glomerular endothelial damage restricting blood flow, leading to acute kidney injury, which in of itself may affect long-term kidney prognosis. Adjusting for SCr could therefore be expected to dampen the effect of TMA on the risk for ESKD since SCr may be on the causal pathway. However, there may be other ways in which TMA affects kidney function without necessarily causing a perceivable rise in SCr. For example, glomerular endothelial damage may have an impact on podocyte function.^19,20^ Interestingly, adjusting just for proteinuria had a minimal effect on the risk for ESKD in our study. Furthermore, the finding of TMA on a kidney biopsy may indicate LN which has simply been causing kidney damage for longer than when there is no TMA. Indeed, similar to SCr, the cases of LN with TMA had more IFTA on biopsy than controls, and when adjusting for the degree of chronicity on biopsy the risk for ESKD was greatly diminished. Although TMA could eventually lead to IFTA, it is more likely that any IFTA already present on biopsy in our study was due to prior LN activity and less likely from the TMA itself. Chronic lesions on biopsy are well-recognized risk factors for progression to kidney failure in any type of kidney disease.^21^ Kidney TMA in LN may therefore be a marker for kidneys that have incurred more irreversible damage and might signal LN presenting at a more advanced stage, thus leading to adverse outcomes. Our study findings propose that it may not be the TMA in of itself that negatively impacts prognosis, as others have also suggested.^10^ A finding of TMA in LN with otherwise preserved GFR and not much chronicity on biopsy (a common finding in patients with LN given their young age) may not necessarily portend a worse prognosis. As pathologists are getting better at recognizing TMA on kidney biopsies, TMA may be a minor contributor to the overall picture, highlighting the importance of considering the whole pathological description of the biopsy. That being said, the sample size in our study led to wide confidence intervals making it difficult to completely rule out an independent effect of kidney TMA on long-term prognosis in LN.

Our cases had shorter follow-up than controls, which may have impacted our findings. Most cases had a more recent index date (46% between 2011 and 2020 compared to 32% for controls) and 3/13 progressed to ESKD within 6 months. This raises the possibility of an era effect dampening the risks for adverse outcomes in our cases, where more cases were diagnosed and treated in the modern era, and thus may have benefited from better care than controls. Indeed, all cases in our study diagnosed in the last decade achieved remission and most did well long term. Another way the shorter follow-up time could have impacted our findings is that if given enough time, more cases could have progressed to ESKD. However, when we restricted our study population to the last decade, yielding more similar follow-up durations, the crude rate of ESKD was not greater in cases than controls.

A sobering observation from our study is the high rates of both cases and controls who had some form of documented non-adherence (59% in all, 54% cases and 61% controls). A systematic review on non-adherence in systemic lupus erythematosus found non-adherence rates ranging from 43% to 75%, similar to what we found.^22^ This is probably one of the main drivers of poor kidney prognosis and ultimately of the high rates of death observed in our study since non-adherence is strongly associated with flaring of disease.^23^ This should serve as a reminder to clinicians that much work needs to be done in properly educating and establishing trust between patients with LN and their medical team.

It is unclear why certain patients with LN develop TMA whereas others do not. Anti-phospholipid syndrome is an important cause of TMA in SLE.^24^ In our study there were similar rates of APLA positivity and of new diagnosis of anti-phospholipid syndrome between cases and controls. Therefore, it is difficult to suggest that the presence of APLA on its own would account for kidney TMA in LN. Another possibility is that, since patients with LN and TMA seem to present with more kidney dysfunction and chronic changes, the presence of TMA may be a delayed manifestation of LN. What is increasingly becoming recognized in all forms of TMA is that, even with a clear triggering event, there often needs to be more than one “hit” for manifestations to arise. An underlying genetic predisposition to TMA due to an overly responsive or inherently active alternative complement pathway from complement protein mutations or deficiencies could be the first “hit”. In LN with kidney TMA, the constant deposition in LN of immune complexes along the glomerular endothelium with ensuing endothelial damage and activation, or the presence of APLA where endothelial activation and coagulation may be mediated by complement,^25^ could represent the second “hit”. This may be why TMA only develops in a minority of individuals with LN and with APLA. One study demonstrated that individuals with TMA and LN had high levels of terminal complement degradation products compared to LN without TMA and that these levels decreased after treatment.^26^ Those with LN and TMA may be inherently predisposed to TMA. Individuals of African descent have been shown to have worse prognosis from LN,^1,2,5^ and such individuals also have greater susceptibility to podocytopathies due to APOL1 risk variants.^27^ Furthermore, the presence of TMA on a kidney biopsy may be associated with podocyte injury and collapsing glomerulopathy.^28,29^ It is interesting to consider what role, if any, APOL1 risk variants may play in the development of TMA or in the progression of scarring caused by TMA in individuals of African descent with LN. This could be a reason why African Americans with LN tend to have worse prognosis compared to other races. This would need to be examined in future studies.

The strengths of our study are that we were able to match our cases with controls based on important demographic and clinical variables, including LN class. We also had granular data in terms of our ability to ascertain remission at 6- and 12-months, something which has not been properly examined in LN with TMA. Our study has important limitations worth discussing. First, the limited sample size led to wide confidence intervals for our risk estimates, making it difficult to draw hard conclusions. We were unfortunately limited by the infrequency of LN with TMA in our registry. Repeating this study by combining LN registries from multiple centers could yield greater sample sizes and help elucidate whether LN with kidney TMA is associated with adverse outcomes regardless of baseline kidney function. This remains an important question since understanding prognostic factors in LN is crucial to guide management and patient counseling, and kidney TMA is readily ascertainable since any patient with LN has had a kidney biopsy. Second, our study was retrospective, with limitations inherent to this design. We did undertake a series of measures to adjust and account for important clinical and pathology variables. Finally, it is a single-center study in a tertiary care setting which receives referrals from throughout the South-Eastern United States. Therefore, our study population may have more severe LN than what may be found in other centers, so results may not be fully generalizable.

## Conclusion

Lupus nephritis with histologic evidence of TMA did not represent disease more refractory to treatment. When present, TMA may be a marker for more severe disease, with a more advanced presentation and which may lead to adverse outcomes, but in of itself TMA without severe presentation does not necessarily portend worse kidney survival compared to LN without TMA. Further studies would be needed to confirm these findings given the limited sample size in our study and in other studies examining outcomes from LN with kidney TMA. Future studies should also look at the role genetic predispositions, such as complement cascade abnormalities or APOL1 risk variants, may be playing in the development of and adverse outcomes from LN with TMA.
